# Agomelatine beyond Borders: Current Evidences of Its Efficacy in Disorders Other than Major Depression

**DOI:** 10.3390/ijms16011111

**Published:** 2015-01-05

**Authors:** Domenico De Berardis, Michele Fornaro, Nicola Serroni, Daniela Campanella, Gabriella Rapini, Luigi Olivieri, Venkataramanujam Srinivasan, Felice Iasevoli, Carmine Tomasetti, Andrea De Bartolomeis, Alessandro Valchera, Giampaolo Perna, Monica Mazza, Marco Di Nicola, Giovanni Martinotti, Massimo Di Giannantonio

**Affiliations:** 1 National Health Service, Department of Mental Health, Psychiatric Service of Diagnosis and Treatment, Hospital “G. Mazzini”, ASL 4, 64100 Teramo, Italy; E-Mail: luigi.olivieri@yahoo.it; 2 Department of Neuroscience and Imaging, University “G. D’Annunzio”, 66013 Chieti, Italy; E-Mails: serroni.nicola@virgilio.it (N.S.); danicampa@virgilio.it (D.C.); gabriella.rapini@aslteramo.it (G.R.); giovanni.martinotti@gmail.com (G.M.); digiannantonio@unich.it (M.D.G.); 3 Department of “Scienze della Formazione”, University of Catania, 95121 Catania, Italy; E-Mail: dott.fornaro@gmail.com; 4 Sri Sathya Sai Medical Educational and Research Foundation, Medical Sciences Research Study Center, Prasanthi Nilayam, 40-Kovai Thirunagar Coimbatore-641014, 641014 Tamilnadu, India; 5 Laboratory of Molecular Psychiatry and Psychopharmacotherapeutics, Section of Psychiatry, Department of Neuroscience, University School of Medicine “Federico II”, 80131 Naples, Italy; E-Mails: felix_ias@hotmail.com (F.I.); carmine.tomasetti@unina.it (C.T.); adebarto@unina.it (A.D.B.); 6 Hermanas Hospitalarias, FoRiPsi, Villa S. Giuseppe Hospital, 63100 Ascoli Piceno, Italy; E-Mail: alessandrovalchera@gmail.com; 7 Hermanas Hospitalarias, FoRiPsi, Department of Clinical Neurosciences, Villa San Benedetto Menni, Albese con Cassano, 22032 Como, Italy; E-Mail: pernagp@tin.it; 8 Department of Psychiatry and Behavioral Sciences, Leonard Miller School of Medicine, University of Miami, Miami, FL 33124, USA; 9 Department of Psychiatry and Neuropsychology, University of Maastricht, 6200 MD Maastricht, The Netherlands; 10 Department of Life, Health and Environmental Sciences, University of L’Aquila, 67100 L’Aquila, Italy; E-Mail: monica.mazza@univaq.it; 11 Institute of Psychiatry and Psychology, Catholic University of Sacred Heart, 00168 Rome, Italy; E-Mail: mdinicola@libero.it

**Keywords:** melatonin, melatonergic receptors, serotonin, dopamine, noradrenaline, agomelatine, anxiety, bipolar depression, seasonal affective disorder, schizophrenia, alcohol dependence, migraines, fibromyalgia, psychiatric disorders

## Abstract

Agomelatine, a melatonergic antidepressant with a rapid onset of action, is one of the most recent drugs in the antidepressant category. Agomelatine’s antidepressant actions are attributed to its sleep-promoting and chronobiotic actions mediated by MT1 and MT2 receptors present in the suprachiasmatic nucleus, as well as to its effects on the blockade of 5-HT2c receptors. Blockade of 5-HT2c receptors causes release of both noradrenaline and dopamine at the fronto-cortical dopaminergic and noradrenergic pathways. The combined actions of agomelatine on MT1/MT2 and 5-HT2c receptors facilitate the resynchronization of altered circadian rhythms and abnormal sleep patterns. Agomelatine appeared to be effective in treating major depression. Moreover, evidence exists that points out a possible efficacy of such drug in the treatment of bipolar depression, anxiety disorders, alcohol dependence, migraines *etc.* Thus, the aim of this narrative review was to elucidate current evidences on the role of agomelatine in disorders other than major depression.

## 1. Introduction

Agomelatine (Valdoxan^®^/Thymanax^®^) (S20098, *N*-[2-(7-methoxynaphth-1-yl)ethyl]acetamide) was first reported in 1992, among a series of synthetic naphthalene melatonin analogs, as acting as an agonist for melatonin MT1 and MT2 receptors and as an antagonist on serotonin 5HT2C receptors [1]. One of the most important pharmacological properties of agomelatine is its pro-chronobiological effect [2]. Various animal models of abrupt shifts and disorganization of the light−dark cycle, of free-running conditions as well as of delayed sleep-phase syndrome have shown that agomelatine accelerates the resynchronization of circadian rhythms of locomotor activity and relevant biological parameters (*i.e.*, body temperature, secretions of hormones) [3,4]. The capacity of agomelatine to synchronize rest−activity rhythms in free-running animals as well as in humans requires the integrity of the suprachiasmatic nucleus (SCN) [5,6].

The accelerating effect of agomelatine was particularly effective if treatment was started three weeks prior to the induced phase shift [7]. Agomelatine treatment did not cause any major change in corticosterone or adrenocorticotropic hormone concentrations, vasopressin, corticotropin-releasing hormone and mineralocorticoid receptor mRNAs levels, which suggests that the mechanism of agomelatine action is not related to hypothalamic–pituitary adrenocortical axis changes [8]. It has also been demonstrated that agomelatine, a potent melatonin receptor agonist drug that strongly binds to and stimulates the activity of melatonin MT1 and MT2 receptors, showed cognitive-enhancing properties, at least in preclinical studies [9].

As specified previously, agomelatine shows agonistic activity with high affinity for melatonin MT1 and MT2 receptors and an antagonist activity with moderate affinity for 5HT2c serotonergic receptor [10]. The 5-HT2c serotonergic antagonism promotes the dopaminergic (DA) firing at the ventral tegmental area, frontal cortex, hypothalamus, hippocampus, medulla and pons, and also the retina—also part of the CNS—via enhancement of norepinephrinergic (NE) activity at the locus coeruleus [11,12]. No significant affinity for any of the monoamine transporters or for adrenergic, noradrenergic, dopaminergic, muscarinic, histaminic and benzodiazepine receptors has been reported [13]. The binding affinity of agomelatine for MT1 and MT2 is similar to melatonin. The literature reported that antidepressant efficacy could be related to melatonin secretion through monoaminergic mechanisms [14].

Moreover, agomelatine has demonstrated the ability to increase adult hippocampal and prefrontal cortex neurogenesis, to enhance expression of brain-derived neurotrophic factor (BDNF), and to trigger several cellular signals, *i.e.*, protein kinase B, extracellular signal-regulated kinase 1/2, and glycogen synthase kinase 3β [15]. Tardito *et al.* [8] suggested that the molecular–cellular effects of agomelatine and, therefore, its antidepressant activity may be the result of a synergistic action between its agonism at MT1/MT2 and antagonism at 5-HT2c receptors. The antidepressant properties of agomelatine related to its effect on neurogenesis, cell survival, BDNF, activity-regulated cytoskeleton associated protein and stress-induced glutamate release, are due to this synergistic action. Moreover, this mechanism of action may explain the superior efficacy of agomelatine on the anhedonia (defined as a loss of interest and lack of reactivity to pleasurable stimuli) [16,17] that may be of particular importance in the treatment not only of major depressive disorder (MDD) with anhedonic features, but also in other neuropsychiatric conditions wherein anhedonia may be present (bipolar depression, anxiety disorders, drug dependence, *etc.*) [18,19,20].

On the other hand, Laudurelle *et al.* [21] showed that, in adult male rats, agomelatine enhanced microtubule dynamics in the hippocampus and to a higher magnitude in the amygdala. By contrast, in the prefrontal cortex, they observed a decrease in microtubule dynamics, whereas spinophilin (a dendritic spines marker) was decreased, and BDNF increased in the hippocampus. Moreover, synaptophysin (presynaptic) and spinophilin were found increased in the prefrontal cortex and amygdala, while PSD-95 (postsynaptic marker) was increased in the amygdala, consistent with the phenomena of synaptic remodeling. These findings may account for the positive effect of agomelatine on cognition.

Agomelatine’s efficacy treatment has been established in several trials and meta-analyses [22,23,24,25,26,27,28]. In November 2008, the committee for medicinal products for human use of the European Medicines Agency provided marketing authorization for treating MDD episodes in adults with agomelatine [29]. On the other hand, recent meta-analyses [30,31,32], summarizing the evidences for agomelatine in the treatment of MDD, reported that the effect sizes were slight and that the efficacy and clinical utility of agomelatine is not uncontroversial.

However, this compound, due to its particular and innovative mechanism of action and the relative lack of side effects [33,34,35], may be a useful option to treat not only MDD, but also several other disorders. Therefore, the aim of this mini-review was to elucidate current evidences on the role of agomelatine in disorders other than MDD.

## 2. Pharmacokinetic Characteristics of Agomelatine

After oral administration, agomelatine is rapidly (Tmax ranging from 0.5 to 4 h) and well absorbed (80%), but its bioavailability is relatively low (<5% at the therapeutic oral dose) due to its high first-pass metabolism [36], which may be of concern especially in elderly patients or in subjects with liver disorders. In humans, agomelatine has a moderate volume of distribution of approximately 35 L, a plasma protein binding of 90%–94% (albumin and alpha 1-acid glycoprotein), and a short plasma half-life (1–2 h) [25]. At the therapeutic levels, agomelatine blood concentration increases proportionally with dose; at higher doses, a saturation of first-pass effect may occur. About 90% of agomelatine is metabolized by cythocrome P450 (CYP) 1A2 (hydroxilation) and about 10% by CYP 450 2C9 (demethylation) isoforms [12]. At higher serum concentrations, also CYP 450 2C19 is involved in the metabolism. Metabolites are conjugated with glucuronic acid and then sulfonated. About 80% of the drug is eliminated through urinary excretion of the metabolites (61%–81% of dose in humans), whereas a small amount of the metabolites undergoes fecal excretion [37].

## 3. Methods

For this review, literature was retrieved through a search of PubMed from 1990 to May 2014, using the medical subject headings (MeSH) of depression, major depressive disorder, bipolar, seasonal depression, anxiety disorders, obsessive−compulsive disorder (OCD), social phobia, panic disorder, generalized anxiety disorder, posttraumatic stress disorder (PTSD), schizophrenia, migraine, alcohol, fibromyalgia, sleep disturbances, suicide, anhedonia, abulia, liver and agomelatine. For additional studies, the author also searched the reference lists of some of the included papers.

## 4. Agomelatine in the Treatment of Bipolar Depression (BDep)

Depression is the prevailing episode of illness in patients with type I (BD-I) or type II bipolar disorder (BD-II) [38]. There has been a paucity of evidence regarding the treatment of BDep, and, in consequence, there is very little consensus regarding recommendations for the management of such patients, often treated with adjunctive antidepressants [39]. To date, only two open-label studies have investigated the efficacy and tolerability of adjunctive agomelatine in BDep.

The first was an open trial conducted in 2007 by Calabrese *et al.* [40], who administered adjunctive agomelatine at 25 mg/day for a minimum of six weeks followed by an optional extension of up to an additional 46 weeks in patients with type I Bipolar Disorder (BD-I) stabilized with lithium (*n* = 14) or valpromide (*n* = 7), and with a Hamilton Rating Scale for Depression (HAM-D) mean score of 25.5 (severely depressed). At the endpoint, 81% of patients met criteria for improvement (>50% improvement from baseline in HAM-D score) and 47.6% responded as early as at one week of treatment. Of the nineteen patients who entered the optional extension period (mean 211 days), eleven completed the one-year extension. No dropouts due to adverse events during the acute phase of treatment (six weeks) was observed, even if six patients experienced serious adverse events during the one-year extension period. Three patients experienced a manic or hypomanic episode during the optional extension period, but only one switch was considered treatment-related.

More recently, Fornaro *et al.* [41] assessed the efficacy and safety of adjunctive agomelatine pharmacotherapy in the treatment of acute major depression in type II bipolar disorder (BD-II). There were 28 subjects with a HAM-D mean score of 25.9 enrolled, 17 (60.7%) taking valproate and 11 (39.3%) taking lithium as their primary mood-stabilizer therapy. A fixed dose of agomelatine (25 mg/day) was administered during the entire study period (6 consecutive weeks as an adjunct to treatment with lithium or valproate, followed by an optional treatment extension of 30 weeks). Using intent to treat analysis, 12 of the 17 valproate-treated (70.6%) and 6 of the 11 (54.5%) lithium-treated patients demonstrated clinical response (defined as >50% decrease in severity from the baseline HAM-D score) to agomelatine augmentation at the six-week primary study endpoint. Six valproate and one lithium-treated patients (25% of the total) responded as early as the second week of treatment. At the 360-week endpoint, 14 of the 17 (82.4%) valproate-treated and 10 of the 11 (90.9%) lithium-treated subjects had a clinical response. Four patients dropped out due to a treatment-related adverse effect by week 6. In the valproate group, these included a single case each of pseudo-vertigo and hypomania; in the lithium group, these included a single case of mania. Two additional cases left the study at week 36 due to hypomania, one each in both the valproate- and lithium-treated groups. During both the short- and longer-term periods of the trial, agomelatine treatment was associated with improvements in depression and sleep quality and was well tolerated.

In conclusion, adjunctive agomelatine may be useful in the treatment of acute BDep, with a good tolerability profile. However, the studies were open-label ones and, although encouraging, must be further confirmed in placebo-controlled or active comparator trials with a longer period of follow up.

## 5. Agomelatine in the Treatment of Seasonal Affective Disorder (SAD)

SAD is a clinical subtype of mood disorder with a typical seasonal pattern (winter depression in which patients experience symptoms of clinical depression during the fall and winter, with full remission to normal mood (or a switch into mania or hypomania) during the spring and summer seasons) [42]. Despite MDD episodes, symptoms of SAD are peculiar and include not only depressed mood, but also fatigue, hypersomnia, hyperphagia with carbohydrate craving and weight gain. Disruption of circadian rhythms have been reported in SAD and a phase shift hypothesis has been suggested [43]. According to this hypothesis, SAD occurs when intrinsic circadian rhythms, such as the melatonin and temperature rhythms, are phase delayed relative to the external clock and/or sleep/wake cycle [44].

Therefore, the agomelatine treatment may be particularly indicated in such a disorder. However, despite this, there is only one open-label study published in 2007. Pjrek *et al.* [45] recruited 37 outpatients (29 women and 8 men) with SAD who received treatment with open-label agomelatine as monotherapy for 14 weeks in a fixed dosage of 25 mg/day. Agomelatine was associated with a statistically significant decrease of symptoms from week 2 onward. Moreover, treatment with agomelatine over 14 weeks yielded a response rate of 75.7% and a remission rate of 70.3% in the intention-to-treat sample. Adverse effects were mild to moderate (mainly sleepiness and fatigue) without dropouts due to side effects and no emerging of manic switch. However, as already statedby the authors’ larger double-blind, randomized, placebo-controlled trials and controlled studies with active comparators (including bright light therapy) are still needed to further define the clinical value of agomelatine as a first-line treatment for SAD.

## 6. Agomelatine in the Treatment of Anxiety Disorders (ADs)

### 6.1. Anxiolytic Properties of Agomelatine

Several studies have demonstrated the anxiolytic properties of agomelatine in animal models [46]. It has been suggested that the anxiolytic effect may be related to the 5-HT2c antagonistic property of such a drug [47]. In fact, it has been demonstrated that mice genetically lacking 5-HT2c receptors showed reduced anxiety, whereas in other experimental models, 5-HT2c receptor agonists show anxiogenic properties [48]. The antagonism of 5-HT2c receptors induced by agomelatine especially in the amygdala and hippocampus may be associated with anxiolytic properties [49] and, always through the blockade of 5-HT2c receptors, agomelatine may also enhance extracellular levels of noradrenaline (NA) in hippocampus, therefore increasing anxiolytic response [50].

Moreover, the anxiolytic effect of agomelatine may also be due to the activation of melatoninergic receptors in response to anxious states [11]. In fact, melatonin secretion is under the facilitatory control of pineal β-adrenoceptors which are innervated by stress-sensitive adrenergic neurons; several stressful and anxiogenic stimuli may enhance pineal release of melatonin [51]. It has been demonstrated that treatment with melatonin reduced pre-operative anxiety [52] and showed anxiolytic properties in mice [53]. Putting these data together, it has been suggested that anxiolysis may be exerted through enhancement of γ-amino butyric acid-related pathways even if this hypothesis needs further confirmation [54].

Efficacy of agomelatine on anxiety disorders (ADs) has become the object of investigation starting from the observation that agomelatine was effective in reducing anxiety symptoms associated with MDD [55].

### 6.2. Generalized Anxiety Disorder (GAD)

To date, there are three published randomized, placebo-controlled trials (RCTs) that evaluated agomelatine efficacy and tolerability in GAD. Of these, two were short-term placebo-controlled studies and one evaluated the long-term efficacy of agomelatine in a relapse prevention trial (Table 1).

Stein *et al.* [56] randomized 121 GAD patients to agomelatine (25–50 mg/day) or placebo in a short-term 12-week study. The results demonstrated a significant superiority of agomelatine 25 to 50 mg than placebo, and the difference between groups was statistically significant in favor of agomelatine from week 6 onward. Agomelatine was as well tolerated as placebo without development of discontinuation symptoms and the most common emergent adverse events were dizziness and nausea.

**Table 1:** Published data on agomelatine treatment of anxiety disorders.

Authors	Reference	Year	Anxiety Disorder	Study Design	Number of Patients	Duration	Dose (mg/day)	Results
Stein *et al.*	[55]	2008	GAD	Randomized, double-blind, placebo-controlled study	121	12 weeks	25 and 50	Agomelatine 25 and 50 mg were statistically more effective than placebo
Crippa *et al.*	[57]	2010	SAD	Case Report	1	6-month follow-up	25	Agomelatine was effective in this SAD patient
Fornaro	[58]	2011	Treatment-resistant OCD	Open-label, case series	6	12-week	50	Agomelatine may have a role in some treatment-resistant OCD patients
Fornaro	[59]	2011	PD	Case report	1	12-month follow-up	25	Agomelatine appeared to be an effective therapy for this PD patient
Da Rocha *et al.*	[60]	2011	Treatment-resistant OCD	Case report	1	3-month follow-up	25	Clomipramine treatment-resistant OCD patients showed clinical improvement with agomelatine
De Berardis *et al.*	[34]	2011	Treatment-resistant OCD	Case report	1	10-month follow-up	50	Agomelatine was effective in this treatment-resistant OCD patient
De Berardis *et al.*	[61]	2012	PTSD	Case report	1	7-month follow-up	50	Agomelatine was effective in the treatment of traffic accident-related PTSD patient
Stein *et al.*	[56]	2012	GAD	Randomized, double-blind, placebo-controlled discontinuation study	227	6-month maintenance period	25 and 50	Agomelatine was superior to placebo in preventing relapse after drug discontinuation
Stein *et al.*	[62]	2014	GAD	Randomized, double-blind, placebo-controlled, parallel group, international, multicenter study	139 in the agomelatine group, 131 in the placebo group and 142 in the escitalopram group	12 weeks	25 and 50	Agomelatine was as effective as escitalopram. Agomelatine was associated with lower incidence of adverse effects than escitalopram

In a later study, Stein *et al.* [62] evaluated the efficacy and tolerability of agomelatine in the prevention of relapse in patients with GAD. Results showed that, during the six-month maintenance period, the proportion of patients who relapsed during the double-blind period in the agomelatine group (22 patients, 19.5%) was lower than in the placebo group (35 patients, 30.7%). The risk of relapse over six months was significantly lower with agomelatine than placebo, and the risk of relapse over time was reduced by 41.8% for agomelatine-treated patients. Moreover, agomelatine was well tolerated without differences in discontinuation symptoms after withdrawal of agomelatine in comparison to maintenance on agomelatine. The most frequent emergent adverse events with agomelatine were similar to those reported during the double-blind treatment period and included headache (11.3%), nasopharyngitis (9.9%), dizziness (8%), nausea (6.5%), dry mouth (5.7%), somnolence (5.0%), and fatigue (4.4%). Fourteen patients treated with agomelatine had at least one emergent and potentially clinically significant abnormal liver enzyme value, but were not discontinued from the study and monitored for liver enzymes.

More recently, Stein *et al.* [63] conducted a 12-week, placebo-controlled, double-blind, randomized, parallel group (*vs.* escitalopram), international, multicenter study aimed to confirm the efficacy of agomelatine 25–50 mg/day in the treatment of patients with a primary diagnosis of GAD. At the endpoint, agomelatine significantly reduced mean Hamilton Anxiety Rating Scale (HARS) total score and showed significant effects also on secondary outcome measures, including psychic and somatic HARS subscales, response rate, remission on the HARS, Clinical Global Impressions–Severity of Illness scale (CGI-S), functional impairment and sleep quality. Interestingly, these findings were confirmed in the subset of more severely ill patients (HARS total score ≥ 25). Agomelatine was well tolerated with no more adverse events than placebo (mainly somnolence, headache, nasopharingitis, and diarrhea), whereas escitalopram was similarly efficacious but was accompanied by a higher incidence of adverse events compared to placebo.

However, despite these positive trials, it should be noted that patients with GAD frequently have comorbid psychiatric and medical illnesses and such trials excluded significant psychiatric and medical comorbidity: therefore, future studies should be extended to, for example, primary-care settings to substantiate generalizability of these results to the general patient population [60].

### 6.3. Other Anxiety Disorders

Agomelatine, on the basis of its capability of restoring circadian rhythms, may be somewhat useful in the treatment of OCD, but data present in the literature are mainly case reports and case series. However, as expected, encouraging evidence emerged when agomelatine was used in the treatment of this disorder as reported by da Rocha and Correa [61], Fornaro [58] and De Berardis *et al.* [59]. Another few published case reports suggest a potential efficacy of agomelatine in panic disorder [57], social phobia [64] and posttraumatic stress disorder [65], but, to date, data are somewhat inconsistent as these observations were sporadic.

## 7. Agomelatine in the Treatment of Partial-Responder Schizophrenia

There is an increasing evidence that suggests that melatonin may play a role in the pathophysiology of schizophrenia and that its modulation in schizophrenia may improve the severity of psychosis [66]. A decreased nocturnal secretion of melatonin has been observed in patients affected by schizophrenia, with the typical diurnal variation in melatonin altered in at least a subgroup of such patients [67]. It has also been reported that monozygotic twins discordant for schizophrenia showed significant alterations in melatonin levels [68]. Interestingly, a noteworthy association between the promoter of the melatonin receptor 1A gene and schizophrenic disorder has been found [69]. Taken together, these data suggest a possible involvement of MT1 and MT2 receptor as potential targets to improve psychotic symptoms and, thus, a favorable action of agomelatine [70]. Moreover, as its 5HT2c receptors’ antagonism may enhance the release of both NE and DA at the fronto-cortical dopaminergic and noradrenergic pathways, indirectly stimulating BDNF release in such areas [71], this may have important implications in the treatment of cognitive symptoms associated with schizophrenia.

In 2014, Bruno *et al.* [72] conducted a 16-week, open-label, preliminary study, on 20 outpatients (9 men and 11 women) with a diagnosis of schizophrenia, who were stable on clozapine monotherapy at the highest tolerable dose (mean dose 430 mg/day) for at least one year. However, according to the Brief Psychiatric Rating Scale (BPRS) total scores >25, patients were considered partial responders to clozapine, due to the presence of residual symptoms. Concerning psychotic symptoms, at week 8, agomelatine augmentation of clozapine significantly improved Positive and Negative Syndrome Scale (PANSS) subscales “negative”, “general psychopathology”, as well as total score and overall clinical symptoms (measured by BPRS). At week 16, significant differences emerged in all PANSS domains, depressive symptoms (measured with the Calgary Depression Scale for Schizophrenia) and overall clinical symptoms. Regarding cognitive performances, at week 8, significant differences emerged only at Wisconsin Card Sorting Test (WCST) “perseverative errors”, whereas, at week 16, agomelatine treatment significantly improved performances on Stroop task and increased improvement on WCST “perseverative errors”. At endpoint, 9 subjects (64.3%) of the 14 completers responded to the coadministration of agomelatine (defined as a reduction in PANSS total score >25% between the baseline and follow-up ratings) and no patients worsened in clinical symptoms *vs.* baseline. The combination of agomelatine−clozapine was generally well tolerated and the most common adverse effects were gastrointestinal symptoms, headache and somnolence.

However, the small sample size, the lack of a control group, the open design, and the non-blinded mode of rating, the lack of clozapine therapeutic drug monitoring in addition to the need of longer-term, randomized, controlled studies do not allow for firm conclusions to be drawn. This may be a starting point for further research in this area as the improvement of cognitive performances in this trial was remarkable and warrants further investigations.

## 8. Agomelatine in the Treatment of the Alcohol-Dependent Associated Sleep Disturbances

It has been suggested that agomelatine may have anti-craving and anti-apathy properties that may be useful in the treatment of substance abuse and dependence [2,10,73]. Moreover, agomelatine is a drug that is not prone to abuse [11,14]. However, no studies were conducted to verify this potentially beneficial action. On the other hand, in a case report published by Grosshans *et al.* [74], the off-label use of agomelatine (up to 75 mg/day) for the treatment of abstinent alcohol-dependent (AHD) associated sleep disturbances was successful. As among ADs patients, sleep disorders are a widespread and persistent problem and may be associated with the risk of alcohol relapse [75]. Grosshans *et al.* [76] expanded their previous observation and treated 8 male and 1 female AHD patients with persistent insomnia but without depression with a flexible dose of agomelatine (up to 50 mg/day) for six weeks, monitoring sleep parameters with the Pittsburgh Sleep Quality Index (PSQI). As most patients had long-term AHD, 7 of 9 patients received either disulfiram (1500 mg/week) or naltrexone (50 mg/day) to prevent relapse. After six weeks of agomelatine treatment, the PSQI global score for all patients decreased significantly. Agomelatine was well tolerated and, remarkably, no increase in serum levels of liver enzymes was observed.

## 9. Agomelatine in the Treatment of Fibromyalgia

Fibromyalgia is characterized by chronic widespread pain, in addition to other symptoms such as fatigue, sleep disturbances, cognitive dysfunction, and psychological distress such as depressive and anxiety symptoms [77]. In fact, depression has been shown to be commonly associated with fibromyalgia and common genetic and pathophysiological mechanisms have been suggested for both diseases [78]. Moreover, sleep disturbances are relatively common in such patients, and are usually described as an unrestorative and poor quality sleep, reflecting a possible disruption in circadian rhythms and melatonin secretion. Melatonin levels are often reduced in fibromyalgia [79], even if this finding is controversial [80]. Melatonin has been successfully used to relieve fibromyalgia symptomatology, with a remarkable improvement on sleep parameters and subjective pain [81,82]. These findings have stimulated researchers to assess the efficacy of agomelatine in such a disease firstly in a positive case report [83].

In a naturalistic prospective open-label pilot study, Calandre *et al.* [84] recruited 23 patients with fibromyalgia and comorbid depressive symptomatology and treated them with flexible agomelatine doses (25–50 mg/day) for 12 weeks. At endpoint, agomelatine therapy was associated with a statistically significant improvement in the Beck Depression Inventory II (BDI-II) total score, even if the effect size was small. Eleven patients (47.8%) were shown to be treatment responders (*i.e.*, when they had at least a five-point drop in the BDI-II score). Concerning secondary outcome measures, both total Fibromyalgia Impact Questionnaire scores and the brief pain inventory severity scores decreased significantly. Surprisingly, the authors did not observe improvement in sleep quality, an unexpected finding given that agomelatine has been shown to improve sleep architecture both in healthy subjects and in depressed patients. A total of 17 patients (74%) experienced treatment-related side effects that were mild and transient. The most frequently reported side effects were dizziness, fatigue, nausea and/or vomiting and insomnia. Two patients withdrew from the study due to adverse events. Authors concluded that agomelatine should not be considered as a first-line option for this condition, but stated that additional studies were needed to evaluate agomelatine’s efficacy in fibromyalgia not associated with clinically relevant depression.

On the other hand, Bruno *et al.* [85] conducted a 12-week, open-label study to further evaluate the efficacy and safety of agomelatine for the treatment of fibromyalgia. They recruited fifteen “drug-free” female subjects, with a mean age of 53.47 years, who met the American College of Rheumatology criteria for primary fibromyalgia. Agomelatine was given at the fixed dose of 25 mg/day until the end of the trial at week 12. They administered rating scales for depression, anxiety, pain and quality of life and assessed sleep disorders. Moreover, they evaluated neurocognitive functioning with the WCST, the Verbal Fluency Task-Controlled Oral Word Association Test and the Stroop Color-Word Test. With regard to clinical symptoms, a statistically significant improvement in pain symptoms, depression and anxiety was observed at week 12. However, no significant differences emerged in the sleep disorders (as assessed by the subitems 4–5–6 of the HDRS) and quality of life. Regarding neurocognitive functioning, the statistical analysis has not detected a significant improvement of the explored dimensions at the end of the observation period, even if a general tendency to a better performance of the sample, in particular in the maintenance of attention during interfering stimuli was noted. The administration of agomelatine was generally well tolerated and only one subject presented adverse effects due to the treatment (headache) which regressed after agomelatine discontinuation.

Although these findings are encouraging, the response to agomelatine treatment was generally less prominent than expected in both studies. In both studies, the sample sizes were small, the observational period short, and the control groups were lacking. In conclusion, since in patients with fibromyalgia the prevalence of comorbid depressive and anxiety disorders is high, it seems quite difficult to disentangle whether the relatively small improvement was due to a direct antidepressant or the unspecific effect of agomelatine.

## 10. Agomelatine in the Treatment of Migraines

Migraines are a primary headache disorder characterized by recurrent episodes of headache associated with gastrointestinal, neurologic, and autonomic symptoms. It may be supposed that the melatonergic system may play a role in the pathogenesis of migraines [86]. In fact, it is well known that the clinical features of migraines may differ considerably, with some patients experiencing headache attacks only at a specific period of the day. In addition, there is a link between migraines and some chronobiological cycles of the human life (e.g., morning migraine, menstrual migraine, weekend migraine) [87]. Moreover, sleep disturbances are consistently associated with chronic headaches [88]. Melatonin has proven to be effective in the treatment of migraines in several studies [89,90,91], even if some studies have failed to confirm these findings [92].

Concerning agomelatine, Tabeeva *et al.* [93] treated for three months with agomelatine 25 mg/day a total of 20 patients with migraines, observing a decrease in migraine attacks as well as in the duration and intensity of such attacks. Moreover, they observed an increase of treatment efficacy index, an improvement of quality of life and duration of disability caused by headaches. Recently, Guglielmo *et al.* [94] reported two cases of patients with migraines successfully treated with agomelatine, one with comorbid depression and the other without comorbidities, both responders to 50 mg/day.

## 11. Safety of Agomelatine

In all reviewed reports, the safety profile of agomelatine, including the incidence of its serious adverse events, was satisfactory. The most common observed adverse effects were headache, dizziness, somnolence, diarrhea, nausea, sedation, fatigue, and insomnia, but all were in the mild-to-moderate range. As the mechanism of action of agomelatine is not associated with increased serotonin levels, its adverse events profile is different from SSRIs and SNRIs, particularly regarding weight gain, headaches, sexual dysfunctions, psychomotor agitation, and serotonin syndrome.

However, hepatotoxic adverse drug reactions with fatal outcome were reported for agomelatine [95]. Clinical studies with agomelatine in MDD did not show clinically significant changes of laboratory parameters, except for elevated liver alanine aminotransferase and/or aspartate aminotransferase (3× upper limit of normal, ULN) in 1.1% of patients treated with agomelatine: even if this adverse effect occurred more often in patients receiving 50 mg of agomelatine daily, it was also observed in those taking 25 mg daily, or those taking placebo [87,96]. In the GAD relapse-prevention study of Stein *et al.* [49], 14 patients treated with agomelatine had at least one emergent and potentially clinically significant abnormal liver enzyme value, but were not discontinued from the study and monitored for liver enzymes. In the subsequent study of Stein *et al.* [50], two patients belonging to the agomelatine group discontinued the study due to treatment-related increase in liver transaminases.

In 2013, the liver toxicity warnings for agomelatine were strengthened in Europe [97]. There had been reports of liver failure resulting in death or liver transplantation. Moreover, it was also warned that agomelatine must not be prescribed when liver function tests are high or to persons of 75 years of age or older [25]. Moreover, in all patients taking agomelatine, liver function must be tested regularly, prior to administration and after 6, 12, and 24 weeks, and even later as clinically indicated [29]. Each patient with elevated transaminase levels should have his/her liver function re-tested in 48 h and the treatment should be discontinued if liver transaminases exceed three times the ULN levels; liver function should be monitored until normal values are re-established [98].

## 12. Conclusions

The unique mechanism of action of agomelatine accounts for its potential benefits in disorders other than MDD [99]. In fact, agomelatine’s sleep-promoting and chronobiotic actions mediated by MT1 and MT2 receptors in the SCN as well as its effects on the blockade of 5-HT2c receptors, are beneficial in several disorders such as BDep, SAD and ADs [100]. Moreover, agomelatine may be potentially useful in the treatment of partial-responder schizophrenia, alcohol-dependent associated sleep disturbances, fibromyalgia and migraines.

However, the majority of evidences suggest that agomelatine may be a potential treatment for ADs (especially GAD) and employed to obtain patient remission rather than merely response (getting the patient 50% better). However, the limited number of studies and their methodological shortcomings should be considered before prescribing agomelatine for ADs. Moreover, the clinical efficacy of this drug will be more evident once it is more widely prescribed and used in the community by physicians. The results of the trials evaluating agomelatine in the treatment of GAD are supportive in regards to its efficacy, and no severe adverse effects were noted. However, to date, the long-term efficacy of agomelatine (>12 months) has not yet been investigated thoroughly; therefore, further long-term studies are needed. Apart from some interesting and positive open-label studies and several encouraging case reports, no randomized or double-blind or placebo-controlled studies are, to date, present in the literature regarding agomelatine in the treatment of disorders other than GAD and MDD. Therefore, the clinical efficacy and the relative good tolerability of such a drug in disorders other than GAD and MDD need further investigations in order to widen the therapeutic spectrum of such disorders.
